# Immune-Enhancing Effect of *Sargassum horneri* on Cyclophosphamide-Induced Immunosuppression in BALB/c Mice and Primary Cultured Splenocytes

**DOI:** 10.3390/molecules27238253

**Published:** 2022-11-26

**Authors:** Hyo In Kim, Dong-Sub Kim, Yunu Jung, Nak-Yun Sung, Minjee Kim, In-Jun Han, Eun Yeong Nho, Joon Ho Hong, Jin-Kyu Lee, Mina Boo, Hye-Lin Kim, Sangyul Baik, Kyung Oh Jung, Sanghyun Lee, Chun Sung Kim, Jinbong Park

**Affiliations:** 1 Department of Surgery, Beth Israel Deaconess Medical Center, Harvard Medical School, Boston, MA 02215, USA; 2 Division of Natural Product Research, Korea Prime Pharmacy Co., Ltd., Suwon 16229, Republic of Korea; 3 Nano Bio Research Center, Jeonnam Bioindustry Foundation, Jangsung 57248, Republic of Korea; 4 Department of Food Regulatory Science, Korea University, Sejong 30019, Republic of Korea; 5 Department of Science in Korean Medicine, Graduate School, Kyung Hee University, Seoul 02447, Republic of Korea; 6 Department of Pharmacology, College of Korean Medicine, Kyung Hee University, Seoul 02447, Republic of Korea; 7 School of Mechanical Engineering, Sungkyunkwan University, Suwon 16419, Republic of Korea; 8 Department of Anatomy, College of Medicine, Chung-Ang University, Seoul 06974, Republic of Korea; 9 Department of Plant Science and Technology, Chung-Ang University, Anseong 17546, Republic of Korea; 10 Department of Oral Biochemistry, College of Dentistry, Chosun University, Gwangju 61452, Republic of Korea

**Keywords:** *Sargassum horneri* (Turner) C. Agardh, immune enhancement, innate immunity, cyclophosphamide, splenocytes, natural killer cells

## Abstract

*Sargassum horneri* (SH) is a seaweed that has several features that benefit health. In this study, we investigated the immune-enhancing effect of SH, focusing on the role of spleen-mediated immune functions. Chromatographic analysis of SH identified six types of monosaccharide contents, including mannose, rhamnose glucose, galactose xylose and fucose. SH increased cell proliferation of primary cultured naïve splenocytes treated with or without cyclophosphamide (CPA), an immunosuppression agent. SH also reversed the CPA-induced decrease in Th1 cytokines. In vivo investigation revealed that SH administration can increase the tissue weight of major immune organs, such as the spleen and thymus. A similar effect was observed in CPA-injected immunosuppressed BALB/c mice. SH treatment increased the weight of the spleen and thymus, blood immune cell count and Th1 cytokine expression. Additionally, the YAC-1-targeting activities of natural killer cells, which are important in innate immunity, were upregulated upon SH treatment. Overall, our study demonstrates the immune-enhancing effect of SH, suggesting its potential as a medicinal or therapeutic agent for pathologic conditions involving immunosuppression.

## 1. Introduction

Seaweed, or macroalgae, refers to thousands of species of macroscopic, multicellular marine algae [1]. Some types of macroalgae contain abundant nutrients, including polyphenols, polysaccharides, proteins and vitamins. Some species of seaweed exhibit potentially beneficial bioactivities, therefore attracting interest in the research community and industry [2].

Brown algae such as Fucus, Laminaria, Sargassum and Undaria spp. contain fucoidan, a high-molecular-weight polysaccharide containing sulfate groups [3]. Fucoidan is a group of marine sulfated polysaccharides from the cell wall matrix of brown algae containing large proportions of L-fucose, sulfate-fucose and galactose, together with minor sugars [4]. Fucoidan-related studies have mainly investigated anticancer [5,6], antiviral [7,8], anti-inflammatory [9,10], anticoagulation, antithrombotic [11] and immune activities [12], which are closely related to molecular weights and sulfate contents [13].

*Sargassum horneri* (Turner) C. Agardh (SH) is a brown alga that is considered an environmental pollutant, especially on Jeju Island, Korea, owing to its large quantities [14]. Large amounts of SH pollute fish farms and cause unpleasant odor at beaches [15]. Furthermore, there is a high economic burden associated with solving such problems [16]. Therefore, the Korean government and researchers have made effort to convert SH into a useful material, for example, use in the construction [17] and livestock industries [18]. Recent efforts have elucidated its beneficial effects in several disease models have. Previous studies have shown that SH has anti-inflammatory [19,20,21], anticancer [22,23], antioxidant [24], immune-enhancing [25] and anticoagulant [26] effects. SH is now widely considered a promising material for health improvement. In a previous study, we showed that SH can promote immune responses in macrophages [27]. However, previous studies only showed an activation or increase in immune responses and did not investigate the beneficial effect of SH on immunosuppression. Additionally, its effect on other immune cells besides macrophages, particularly neutrophils, T cells and natural killer (NK) cells, is yet to be determined.

The immune system is composed of the innate immune and adaptive immune systems [28]. Innate immunity works as the immediate responder and first-line defender against external pathogens through the activities of macrophages, dendritic cells, monocytes, neutrophils and NK cells [29]. The adaptive immune system creates a whole-body defense network by training immune cells to remember such external antigens. This network is regulated through the activation of immune B and T cells against antigens and is regulated by immune globulins, chemokines and cytokines secreted by various immune cells [30]. The immune system defends the body from outside pathogens such as bacteria, fungi and viruses. Pathological dysfunction of either immune system may cause a serious health risk by failing to prepare the body to fight against pathogens, leading to various diseases, such as infection and cancer [31].

In this study, we investigated the effect of SH on immune functions, focusing on spleen-related immune cells. Immune function tests were performed in primary cultured splenocytes, which are among the most abundant supplies of immune cells. We also used in vivo models. First, we studied the effect of SH on immune-related parameters in naïve mice; then, we used an in vivo immunosuppression model by injecting cyclophosphamide (CPA) and verified the immune-enhancing effect of SH.

## 2. Results

### 2.1. Total Sugar and Sulfate Group Contents of SH

Although no clearly certified analysis method is available to quantify fucoidan, purity is generally calculated as the sum of the total sugar and sulfate groups [32]. The contents of total sugar and sulfate groups of SH were measured in this study. The total sugar contents were 39.69%, and the sulfate group contents were 16.93% (Supplementary Table S1). Because polysaccharides in seaweed are known to exert several beneficial effects [33,34], high total sugar contents are assumed to serve as an indicator of an effective process for the extraction of diverse functional substances from seaweed.

### 2.2. Monosaccharide Composition of SH

**Figure 1:**
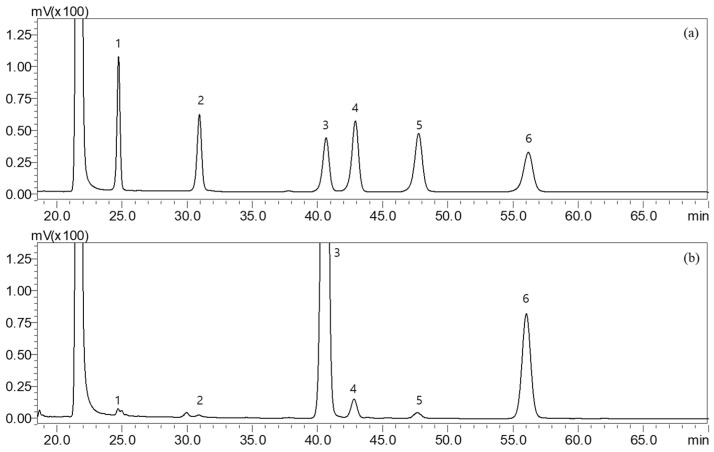
HPLC chromatograms of PMP-derivatized monosaccharides. Peaks in (**a**) mixed standard and (**b**) hot-water extract of SH were analyzed by HPLC. Peak #1. mannose; 2. rhamnose; 3. glucose; 4. galactose; 5. xylose; 6. fucose.

Fucoidan is a sulfate polysaccharide mainly composed of fucose-repeating units, in as well as galactose, mannose, glucose and glucuronic acid [35]. As a heterogeneous polymer, fucoidan exhibits considerable structural diversity, which makes it difficult to draw general conclusions [36]. Fucoidans have traditionally been quantified colorimetrically with hydrochloric acid and homocysteine sulfuric acid. Gas chromatography (GC) has also been used to determine L-fucose in fucoidans. In this study, the composition of monosaccharides was confirmed using high-performance liquid chromatography (HPLC). The monosaccharide composition was analyzed after hydrolyzing the hot-water extract powder of SH and derivatizing the hydrolysates with 1-phenyl-3-methyl-5-pyrazolone. Results are shown in Figure 1 and Table 1. Fucose was identified as the main component, accounting for 59.49 mg/g of the extract, and galactose comprised 8.10 mg/g. In studies on other types of brown algae, *Fucus vesiculosus* was reported to be composed of 83.1% fucose, 7.3% galactose, 6.5% xylose and 2.0% mannose [37], and *Fucus distichus* was reported to be composed of 51.6% fucose, 1.5% galactose, 2.7% xylose, 0.7% mannose and 0.2% glucose [38]. The contents of fucose, a monosaccharide that is a component of fucoidan, were reported to be 24–40% by Fletcher et al. [39] and 35.5–67.8% by Duarte et al. [40], with slight differences in the composition of monosaccharides and fucose content depending on the seaweed.

### 2.3. Molecular Weight Distribution

**Figure 2:**
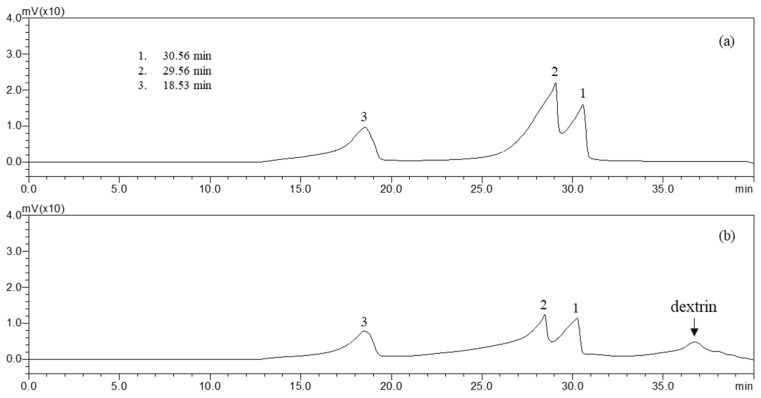
GPC chromatogram of molecular weight. Peak in (**a**) SH hot-water extract and (**b**) SH hot-water extract after spray drying (dextrin added).

The molecular weight (MW) of fucoidan is an important parameter affecting biological activity [5]. Low-MW fucoidan exhibits more antioxidant activity than high-MW fucoidan. High-performance gel permeation chromatography (HP-GPC) results obtained in this study (Figure 2) indicate that SH contains a high-MW fraction and a low-MW fraction. Compared with the pullulan standard elution curve of Shodex standard P-82, the major high-MW fraction peak (1) was about 64.1 to 70.8 × 10^4^, whereas the minor low-MW fraction peak (2) was 1.1 to 1.2 × 10^4^, and the small fraction peak (3) was 0.6–0.7 × 10^4^.

### 2.4. SH Enhances Immune Function in CPA-Induced Immunosuppressed BALB/c Mice

**Figure 3:**
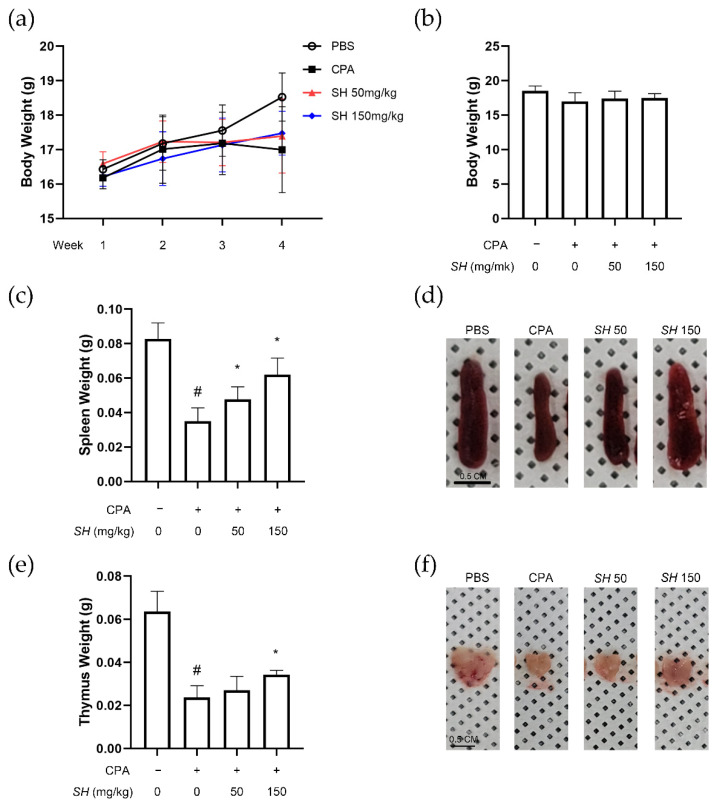
Effects of SH on immune function in a CPA-induced immunosuppressed in vivo model. SH was orally administrated in BALB/c mice for 14 days. CPA was injected on days 14 and 16 to induce immunosuppression. After sacrifice, immune-related parameters were measured. (**a**,**b**) The body weight of each group was measured, as well as the issue weight and size of (**c**,**d**) spleen tissues and (**e**,**f**) thymus tissues. # *p* < 0.05 vs. naïve mice, * *p* < 0.05 vs. CPA-injected mice. Results are displayed as mean ± SD of *n* = 10 per group.

For a pilot study, we treated the normal immune system of naïve mice with SH. SH altered the immune system by increasing the spleen size, splenocyte proliferation and NK cell activity (Supplementary Figure S1). Next, to determine whether SH can restore the immune function suppressed by CPA, we constructed an immunosuppression model by intraperitoneally injecting mice with CPA. CPA injection resulted in a slight decrease in body weight, but SH administration protected from excessive body weight loss. All changes were nonsignificant (Figure 3a,b). Average tissue weight and size of the spleen (Figure 3c,d) and thymus (Figure 3e,f) were decreased by CPA injection but recovered in the SH-treated mice.

We also observed increased T-cell proliferation. Flow cytometry analysis could confirm that SH treatment induces the expression of CD3^+^/CD25^+^ T cells. In particular, significantly increased CD4^+^/CD25^+^ expression indicated that the number of CD4-positive helper T-cells was increased in the SH-fed group. The number of CD3^+^/CD25^+^ T-cell was 346 ± 23 cells in the control group, which was reduced to 296 ± 9 cells in the CPA group. *SH* treatment increased the number of these T-cells to 340 ± 19 cells (50 mg/kg) and 373 ± 22 cells (150 mg/kg), respectively (Supplementary Figure S2a). Similarly, the number of CD4^+^/CD25^+^ T cells was decreased from 676 ± 32 cells to 389 ± 25 cells by CPA injection. This effect was reversed by *SH* administration: 419 ± 28 cells with 50 mg/kg and 506 ± 7 cells with 150 mg/kg. Cell numbers were counted, revealing a total of 10,000 cells of isolated splenocytes (Supplementary Figure S2b). We next evaluated the number of neutrophils in the spleen with CD11b and Ly6G. Among 10,000 total cells, 1590 neutrophils were identified. This number was decreased by 31% upon CPA treatment (1100 cells). In *SH*-treated mice the number of neutrophils was 1400 and 1350 with 50 mg/kg and 150 mg/kg doses, respectively (Supplementary Figure S2c).

**Figure 4:**
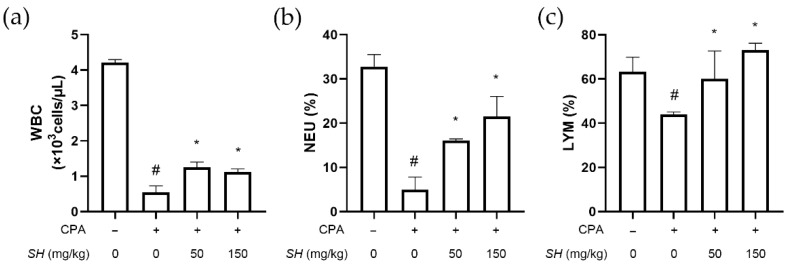
Effects of SH on immune cell count in a CPA-induced immunosuppressed in vivo model. SH was orally administrated in BALB/c mice for 14 days. CPA was injected on days 14 and 16 to induce immunosuppression. After sacrifice, blood was collected, and the immune cell count of (**a**) total WBC, (**b**) neutrophils (NEU) and (**c**) lymphocytes (LYM) was measured. # *p* < 0.05 vs. naïve mice, * *p* < 0.05 vs. CPA-injected mice. Results are displayed as mean ± SD of *n* = 10 per group.

Analysis of the blood component parameters showed that SH treatment increases the CPA-induced loss of immune cells. Figure 4 shows that SH treatment revoked CPA-induced changes in white blood cell (WBC) count, neutrophil ratio and lymphocyte ratio. CPA also decreased the number of red blood cells (RBCs) and hemoglobin content, but these blood parameters were restored to normal levels by SH treatment (Table 2).

### 2.5. SH Induces Proliferation of Splenocytes, NK Cell Activity and Th1 Cytokine Expression In Vivo

**Figure 5:**
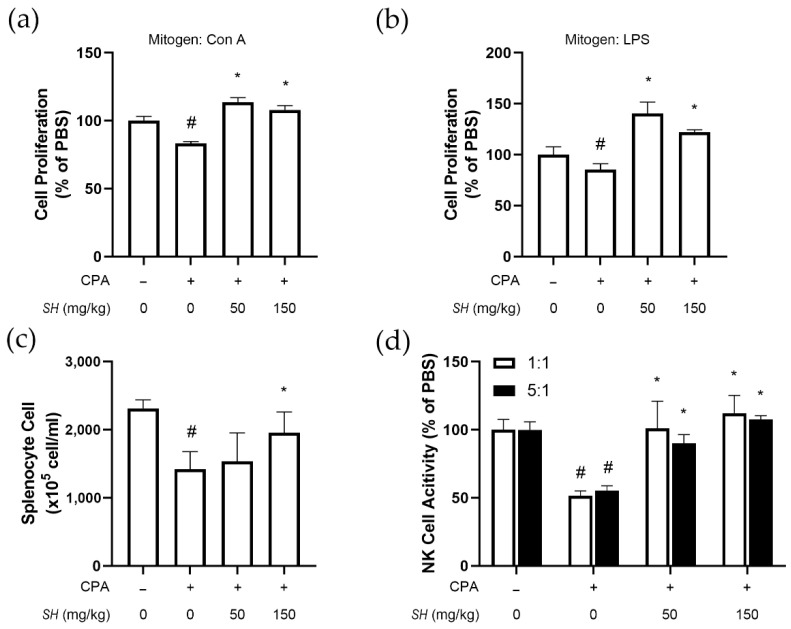
Effects of SH on immune functions of splenocytes isolated from CPA-induced immunosuppressed mice. SH was orally administrated in BALB/c mice for 14 days. CPA was injected on days 14 and 16 to induce immunosuppression. After sacrifice, splenocytes were isolated, and (**a**) splenocyte numbers and proliferation in response to (**b**) Con A or (**c**) LPS were measured. (**d**) NK cells were purified from splenocytes, and YAC-1-targeting activity was measured. # *p* < 0.05 vs. splenocytes from naïve mice, * *p* < 0.05 vs. splenocytes from CPA-injected mice. Results are displayed as mean ± SD of three or more separate experiments.

**Figure 6:**
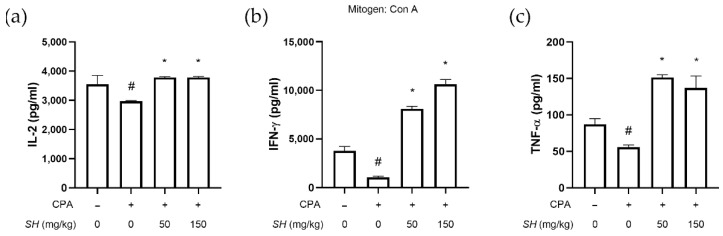
Effects of SH on cytokine expression in a CPA-induced immunosuppressed in vivo model. SH was orally administrated in BALB/c mice for 14 days. CPA was injected on days 14 and 16 to induce immunosuppression. After sacrifice, blood was collected, serum was separated, and cytokine expression of (**a**) IL-2, (**b**) IFN-γ and (**c**) TNF-α was measured using the ELISA method. # *p* < 0.05 vs. naïve mice, * *p* < 0.05 vs. CPA-injected mice. Results are displayed as mean ± SD of three or more separate experiments.

Next, we investigated the function of immune cells of SH-fed mice. Splenocytes were harvested from the control group, CPA-injected group and CPA-injected and SH-fed groups. We found that CPA injection decreased the number of splenocytes, but SH treatment, especially at a higher dose, reversed the decrease (Figure 5a). In these cells, either Con A (Figure 5a) or lipopolysaccharide (LPS) (Figure 5b) was treated, and the proliferation of splenocytes was evaluated. As shown in Figure 5d, SH treatment also increased the activity of CPA-suppressed NK cells, both in 1:1 and 5:1 ratios of incubation with YAC-1 cells. Concomitant to the in vitro results, CPA repressed the cytokine expression of interleukin-2 (IL-2), interferon gamma (IFN-γ) and tumor necrosis factor alpha (TNF-α); however, SH treatment significantly reversed this decrease (Figure 6).

### 2.6. SH Induces Proliferation of Splenocytes

**Figure 7:**
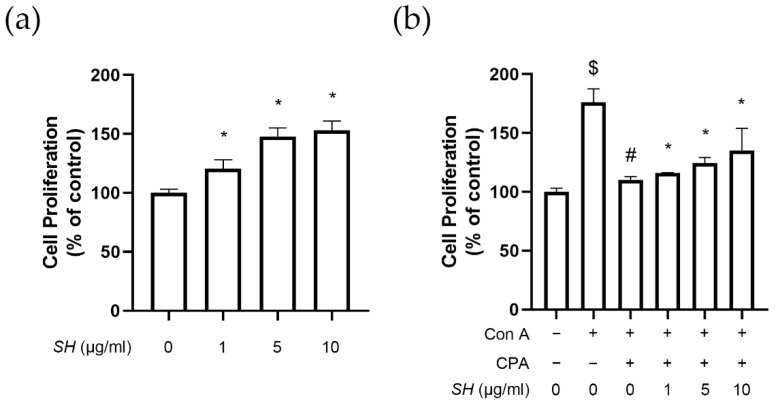
Effects of SH on splenocyte proliferation. Splenocytes were isolated from spleen tissues of naïve mice. (**a**) The cell proliferation of splenocytes measured after 24 h treatment with SH. * *p* < 0.05 vs. untreated cells. (**b**) Immunosuppression in splenocytes was induced with CPA treatment for 24 h. Then, cell proliferation of splenocytes was measured after 48 h treatment with Con A with or without SH. $ *p* < 0.05 vs. untreated cells, # *p* < 0.05 vs. Con A-treated cells, * *p* < 0.05 vs. Con A/CPA-treated cells. Results are displayed as mean ± SD of three or more separate experiments.

To assess the immune-enhancing effect of SH, we used the primary cultured splenocyte model. The spleen is one of the most important members of the whole-body immune system, and splenocytes consisted of various immune cells, such as T and B lymphocytes, dendritic cells and macrophages. SH treatment induced the proliferation of splenocytes (Figure 7a). Next, as shown in Figure 7b, we used the broad immune-suppressing agent CPA. When Con A was treated as the mitogen, increased proliferation was observed; however, when CPA was treatment was combined with Con A, this proliferation was reduced to the basal level. SH resulted in a dose-dependent increase in cell proliferation when added to the CPA- and Con A-cotreated splenocytes.

### 2.7. SH Increases Th1 Cytokine Expression in Splenocytes

**Figure 8:**
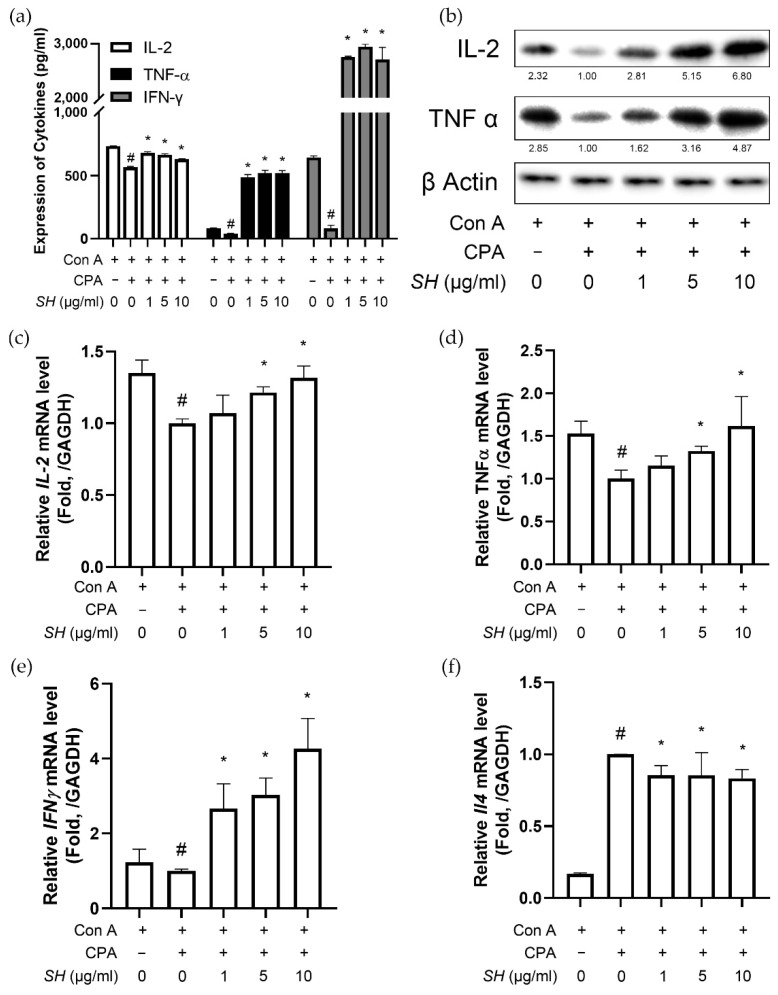
Effects of SH on cytokine expression in splenocytes. Splenocytes isolated from naïve mice were treated with various concentrations of SH. Then, the cytokine expression of (a) IL-2, TNF-α and IFN-γ was measured using the ELISA method. (**b**) Protein levels of cellular IL-2 and TNF-α were measured by Western blotting. mRNA levels of (**c**) *Il2*, (**d**) *Tnfa*, (**e**) *Ifng* and (**f**) *Il4* were measured by real-time RT-PCR. # *p* < 0.05 vs. Con A-treated cells, * *p* < 0.05 vs. Con A/CPA-treated cells. Results are displayed as mean ± SD of three or more separate experiments.

Next, we analyzed the cytokine levels. IL-2 is a cytokine that regulates leukocyte and lymphocyte activities [41]. IFN-γ involves immune surveillance by establishing appropriate adaptive immunity [42]. The cytokine TNF-α is a major regulator of inflammatory responses primarily secreted by macrophages [43]. Whereas CPA treatment suppressed the expression of these cytokines in splenocytes, SH successfully restored cytokine expression of IL-2, IFN-γ and TNF-α (Figure 8a).

We then measured the cytokine expression in the isolated splenocytes. Previous reports suggest that CPA causes a pathological imbalance between Th1 and Th2 cytokines [44]. As shown in Figure 8, CPA suppressed the genes that transcribe Th1 cytokines (*Il2*, *Tnfa* and *Ifng*) and increased the mRNA level of *Il4* (a gene for Th2 cytokine [45]). SH treatment reversed this change, as the SH group showed increased IFN-γ and decreased *Il4* expressions compared to the Con A/CPA-treated splenocytes (Figure 8). Moreover, the ratio of Th1-to-Th2 cytokine gene expression was increased by SH treatment, suggesting a recovery of the Th1/Th2 imbalance caused by CPA.

## 3. Discussion

SH is a seaweed used as a medicinal food rich in nutrition and mostly consumed in China, Japan and Korea [19]. Although it had been long considered useless waste in the sea, recent attention from the Ministry of Food and Drug Safety of Korea (MFDS) led to the discovery of the considerable potential of this seaweed in terms of health-beneficial functions [46]. SH is rich in fucoidan, a cell wall polysaccharide obtained from brown algae, also known as fucose-containing sulfated polysaccharide (FCSP). L-fucose dominates other monosaccharides, such as galactose, mannose, glucose and uronic acid. SH is a resource rich in fucoidan, with high fucose content compared to other seaweeds in the Sargassum genus [47].

For instance, SH has an anti-allergic effect in type I allergic responses; Han et al. showed that SH reduced the PCA reaction in an IgE/BSA-induced type I allergic mouse model [46]. The same group also demonstrated its antiatopic effect in NC/Nga mice [48]. Its anti-inflammatory effect, in particular on fine-dust- or particulate-matter-induced inflammatory responses, has also been reported [20,21,49]. SH can attenuate oxidative action and neuroinflammatory responses [24] and has shown beneficial effects in several metabolic diseases, such as obesity, diabetes and hepatic steatosis [50]. Anticancer effects of its polysaccharides have also been described [22,23]. In this study, we investigated the immune-enhancing effects of SH in immunocompromised mice and splenocytes.

The immune system is one of the most important functions of the human body. Without proper immune functions, i.e., under immunosuppressed conditions, the ability of the immune system to fight against infection is diminished, representing a critical health threat. Most cases of immunosuppression are acquired, resulting from extrinsic factors affecting the immune system. CPA is an alkylating agent primarily used to treat various types of cancers, including multiple myeloma, sarcoma and breast cancer [51]. As CPA has been used for more than four decades, clinical experience has demonstrated its potential as an immunosuppressive agent for the treatment of autoimmune and immune-mediated diseases [52]. CPA acts as a potent immunosuppressive agent, affecting T cells and B cells to decrease immune responses by blocking the production of DNA [53]. Because CPA can inhibit both humoral [54] and cellular [52] immune responses, its application in vivo is now accepted as an experimental model for immunosuppression [55]. CPA also affects the activity of NK cells [56]. Here, we show that oral administration of SH in mice altered the immune system, especially under conditions of CPA-induced immunosuppression, by inducing proliferation of splenocytes, upregulating related cytokines and increasing NK cell activities.

NK cells are lymphocytes that participate in early defense and immune responses against foreign pathogens and autologous cells undergoing stress in various forms, such as microbial infection or tumor transformation [57]. Whereas adaptive immunity governed by T cells and B cells provides the host with prolonged specific defense against pathogens, the first line of immune function mostly depends on the innate immune system, comprising neutrophils, macrophages and NK cells. This is well-described in patients with defective innate immunity, which are highly susceptible to uncontrolled, fatal infections [58]. The name NK cell comes from its functional ability to kill cancer cells without any sensitization. Therefore, NK cells are considered a component of innate immunity. Additionally, activated NK cells release and secrete cytolytic granules or inflammatory cytokines, which can activate and recruit components of both the innate and adaptive immune response [59]. Recent advances in NK cell research revealed its participation in adaptive immunity [60]. Our results demonstrate that SH can increase NK cell activity, as demonstrated in normal mice fed SH, as well as in mice with CPA-induced immunosuppression.

T lymphocytes are the main source of cytokines [61]. T cells bear antigen-specific receptors on the cell surface, which allow for recognition of pathogens. These receptors are described as a cluster of differentiation (CD). T cells require CD3 for binding and activation [62]. CD25 is used to determine the activation of T cells, as it acts as the receptor of IL-2, which regulates T-cell differentiation [63]. T cells are categorized into two main subsets: those expressing CD4 are known as helper T cells, and CD8-expressing cells are called killer T cells. Whereas killer T cells display cytotoxicity to pathogens, helper T cells are known as cytokine producers [64]. Helper T cells are further subcategorized into Th1 and Th2 depending on the cytokines they express, i.e., Th1-type cytokines or Th2-type cytokines. Th1 cytokines stimulate other immune cells, such as macrophages, lymphocytes and neutrophils, when required [65]. Th1-type cytokines produce proinflammatory responses via secretion of Th1 cytokines, including IL-2, IFN-γ and TNF-α [66]. IFN-γ is an important regulator of macrophages, which are secreted by immune cells, such as T cells, macrophages and NK cells. It is also recognized as a representative marker of Th1 T cells. IL-4 is a differentiation factor that induces the differentiation of Th0 cells to CD4^+^ Th2 cells [45]. In an ideal immune system, Th1 and Th2 cells actively interact to create a balance through a complementary relationship [67]. Our results show that SH treatment induced the expression of Th1 cytokines, implying a significant immune-enhancing effect of seaweed. Furthermore, neutrophils were increased by *SH* treatment, likely stimulated by Th1-related responses.

Overall, the results of this study demonstrate that SH can work as a powerful immune-enhancing agent and can be used as a therapeutic or functional food for patients with a repressed immune system.

## 4. Materials and Methods

### 4.1. Sample Purchase and Extract Preparation

The raw materials of the SH used in this study were collected from Korea. To reduce moisture content to less than 10%, collected SH was dried through an immersion washing and boiling process using purified water. To prepare hot-water extracts of SH, a dried sample was cut into appropriate sizes, added to 40 times of purified water to extract the content for 4 h at 90 °C. The extract was filtered using a 55 μm bag filter, and titanium dioxide was added to reduce arsenic. Then, the extract was concentrated to 20% brix by a centrifugal thin film evaporator and spray-dried by adding 30% dextrin. The dose of administration used in the study is indicated, excluding the 30% dextrin portion.

### 4.2. Measurement of Total Sugar Contents

The total sugar contents were measured according to the method described by Dubois [68]. After adding 100μL of 6% phenol and 1.5 mL of sulfuric acid to 200 μL of the sample at a concentration of 1.0 mg/mL, the mixture was left to react for 10 min at 95–100 °C and cooled thereafter. The absorbance was measured at 490 nm using a microplate reader. The standard calibration curve was drawn by converting the glucose contents in the standard curve drawn using the same method using glucose as a standard substance into total sugar contents.

### 4.3. Measurement of Sulfate Group Contents

The sulfate group contents were measured with reference to the Dodgson method [69]. After dissolving 5 mL of 6 M hydrochloric acid solution in 0.05 g of the sample, the sample was decomposed for two hours at 95–100 °C. The decomposed sample was cooled and mixed, 0.2 mL of the mixed sample was transferred to a test tube, 3.8 mL of 4% TCA solution and 1 mL of BaCl_2_-gelatin reagent were added, the mixture was stirred for 20 min and the absorbance was measured at 360 nm using a microplate reader. The standard calibration curve was drawn by converting the Na_2_SO_4_ contents in the standard curve drawn after conducting the same experiment using Na_2_SO_4_ as a standard substance into sulfate group contents. The BaCl_2_-gelatin reagent was prepared by heating 1 g of gelatin in 200 mL of distilled water at 65 °C. The gelatin was left unattended at 4 °C overnight, and 1 g of BaCl_2_ was added to dissolve the gelatin. The resulting BaCl_2_-gelatin reagent made was left unattended for 2–3 h before use.

### 4.4. Measurement of Monosaccharide Contents

The monosaccharides contents were measured with reference to the method described by Hong et al. [70]. In brief, 0.2 g of freeze-dried extract was weighed directly into a 50 mL glass vial, and 10 mL of 2 M HCl solution was added. Then, SH was hydrolyzed in boiling water at 90 °C for 1 h and cooled. The hydrolyzed sample was transferred to a 50 mL conical tube and neutralized to pH 7 by adding an appropriate amount of 2 M NaOH. The neutralization solution was diluted to 50 g with ultrapure deionized water and filtered with a syringe filter (0.45 μm, PTFE). For the derivatization of sugar, 2 mL of acid hydrolyzate was added to a 10 mL glass vial, and 2 mL of 0.5 M PMP solution and 2 mL of 0.3 M NaOH were added. The reaction was induced in a water bath heated to 70 °C for 30 min and then cooled to room temperature. Thereafter, 2 mL of 0.3 M HCl was added, and the mixture was vortexed for about 1 min until a solid precipitate was formed. It was filtered through a syringe filter (0.45 μm, PTFE) and transferred to a 15 mL conical tube, and 4 mL of chloroform was added. The conical tube was shaken and centrifuged for 10 min at 10 °C and 3000 rpm, and the supernatant in the aqueous phase was obtained and analyzed using HPLC.

### 4.5. HPLC-Diode Array Detector (DAD) Analysis

HPLC was conducted using Shimadzu liquid chromatography system (LC-2AD) with a diode array detector (SPD-M20A) and a Shim-pack Synergi^TM^ 4 μm Polar-RP 80 Å (250 mm × 4.6 mm I.D.) at 35 °C. The mobile phase consisted of two positions (solvents A and B). Solvent A was distilled water with 0.1 M ammonium acetate (pH 5.0 adjusted for acetic acid), and solvent B was acetonitrile. The gradient was 0–5.0 min, 10% B; 5.0–10.0 min, 10–20% B; 10.0–65.0 min, 20% B; 65.0–65.1 min, 20–95% B; 65.1–70.0 min, 95% B; 70.0–70.1 min, 95–10% B; 70.1–77.0 min, 10% B. The run time was 77 min, with a flow rate of 1.0 mL/min. The UV detection wavelength was 245 nm, and peaks were identified by comparing their relative retention times with those of authentic standards analyzed under the same conditions. The monosaccharide standards were derivatized by the same method as the sample. All reagents were purchased from Sigma-Aldrich (St. Louis, MO, USA).

### 4.6. Molecular Weight Distribution

The molecular weight distribution of the fucoidan was measured by high-performance gel permeation chromatography (HP-GPC). A Shimadzu liquid chromatography system (LC-20AD) was used, including a refractive index detector (RID-20A) and a GS-520 HQ + GS-320 HQ (300 mm × 7.5 mm I.D., 7 μm) (Shodex Asahipak, Tokyo, Japan). The column was eluted isocratically with 30% MeOH at 40 °C and a flow rate of 0.5 mL/min. Standard pullulan (Shodex Standard P-82 kit) with an average molecular weight (Mw) ranging from 0.60 to 78.8 × 10^4^ was diluted to 0.5%, and the injection volume was 30 μL.

### 4.7. Animal Experiments

Five-week-old BALB/c mice were purchased from Seronbio (Gyeonggi-do, Uiwang-si, Korea) and maintained for 1 week before the experiments. For the immune-enhancing study, mice were randomly divided into four groups after acclimation (*n* = 5 per group). The mice were orally administrated PBS or SH at doses of 50, 100 and 150 mg/kg/d for 14 consecutive days. For the immunosuppression model study, five-week-old BALB/c mice (Seronbio, Uiwang-si, Gyeonggi-do, Republic of Korea) were maintained for 1 week for acclimation and then divided into four groups as follows (*n* = 10 per group): vehicle group treated with PBS, CPA-injected group, CPA-injected and SH (50 mg/kg/d)-fed group, and CPA-injected and SH (150 mg/kg/d)-fed group. PBS or SH was orally administered once a day for 14 consecutive days. To induce immunosuppression, BALB/C mice were intraperitoneally injected with CPA twice (150 mg/kg on day 14 and 100 mg/kg on day 16). Animals were sacrificed on day 21, and the spleen and thymus were isolated. All animal experiments were conducted according to the university guidelines of ethical committee for Animal Care and ChemOn Inc. (Gyeonggi-do, Yongin-si, Republic of Korea) protocol (approval number CHEM-2022-IA0064-00).

### 4.8. Isolation of Splenocytes

After mice were sacrificed, spleen tissues were harvested and placed in Roswell Park Memorial Institute (RPMI) 1640 medium (Invitrogen, Carlsbad, CA, USA). For the isolation of splenocytes, and the spleens were homogenized using a Biomasher II (Biomasher, Tokyo, Japan). Samples were spun down by centrifugation at 1200 rpm for 5 min. In order to lyse the erythrocytes, the pellets were resuspended in red blood cell lysing ACK lysing buffer (Gibco, Grand Island, NY, USA), incubated for 1 min and centrifuged at 1200 rpm for 5 min. Splenocytes were passed twice through a cell strainer (40 μm nylon, Corning, Corning Inc., New York, NY, USA) in RPMI 1640. Then, after being spun down, pellets were resuspended in RPMI 1640 medium containing 10% FBS, and the number of cells/mL was measured using a hemacytometer after staining with trypan blue solution.

### 4.9. Splenocyte Cytotoxicity and Proliferation Assay

The in vitro proliferative effects of SH on splenocytes were evaluated using a cell proliferation 3-(4,5-dimethylthiazol-2-yl)-5-(3-carboxymethoxyphenyl)-2-(4-sulfophenyl)-2H-tetrazolium (MTS) kit (Promega, Madison, WI, USA). Briefly, primary cultured splenocytes were distributed in a 96-well plate (5 × 10^6^ cells/mL) and treated with SH (0, 1, 5 and 10 μg/mL) for 24 h. Absorbance at 540 nm was measured using a microplate reader (Bio Teck, Winooski, Vermont, USA). In the CPA-induced immunosuppression in vitro model, splenocytes were incubated in the presence or absence of 1000 μg/mL CPA for 24 h. Then, the cells were treated with Con A (5 μg/mL) and SH (0, 1, 5 and 10 μg/mL) for 48 h, and absorbance was measured.

### 4.10. Flow Cytometry

Cell-surface CD expressions were detected by flow cytometry using a BD FACSCalibur (BD Biosciences, Haryana, India), as described previously [71]. An FITC-conjugated anti-mouse CD3 antibody (Cat# 100203, BioLegend, San Diego, CA, USA), FITC-conjugated anti-mouse CD4 antibody (Cat# 116003, BioLegend) and PE-conjugated anti-mouse CD25 antibody (Cat# 101904, BioLegend) were used.

### 4.11. Analysis of Blood Cell Count and Related Parameters

Total RBC, HGB, hematocrit, mean cell volume, mean cell hemoglobin, mean cell hemoglobin concentration, total WBC, neutrophils, lymphocytes, monocytes, eosinophils and basophils were measured using an automated hematology analyzer (XN-V, Sysmex, Kobe, Japan).

### 4.12. Determination of Cytokines

Cell culture media were collected from splenocytes treated with SH in vitro or in vivo. Levels of IL-2, IFN-γ and TNF-α were quantified in culture supernatants using enzyme-linked immunosorbent assay kits according to the manufacturer’s instructions (Invitrogen, CA, USA).

### 4.13. Determination of NK Activity

To isolate NK cells from splenocytes, we used the magnetic activated cell sorting method with MACS microbeads (Miltenyi Biotec, Gladbach, Germany). Isolated NK cells were added to seeded YAC-1 cells (4.5 × 10^4^ cells/well to 96-well plates) in 1:1 and 5:1 ratios and incubated for 18 h. Then, the NK activity was assessed according to the cytotoxicity of NK cells to NK-sensitive target YAC-1 cells using an EZ-LDH kit (Daeil Lab Science, Seoul, Republic of Korea).

### 4.14. Western Blot Analysis

Protein extracts were prepared by homogenization in radioimmunoprecipitation assay (RIPA) buffer (Cell Signaling Technology, Danvers, MA, USA). Lysates were resolved by sodium dodecyl sulfate (SDS)-polyacrylamide gel electrophoresis and transferred onto polyvinylidene difluoride (PVDF) membranes (Millipore, Darmstadt, Germany). Then, the membranes were blocked and incubated with the indicated primary antibodies (1:1000), followed by incubation with horseradish peroxidase-conjugated secondary antibodies (1:10,000). Protein signals were detected using an ECL advance kit (GE Healthcare Life Sciences, Seoul, Republic of Korea).

### 4.15. RNA Isolation and Real-Time Reverse Transcription-Polymerase Chain Reaction (RT-PCR)

RNA isolation and real-time RT-PCR procedures were performed as previously described [72]. *Gapdh* was used as the endogenous control. The following primers were used in this study:

*Il2* (F: 5′-GCACCCACTTCAAGCTCCA-3′,

     R: 5′-AAATTTGAAGGTGAGCATCCTG-3′)

*Il4* (F: 5′-GGTCTCAACCCCCAGCTAGT-3′,

     R: 5′-GCCGATGATCTCTCAAGTGAT-3′)

*Tnfa* (F: 5′-GAGAAGTTCCCAAATGGC-3′,

     R: 5′-ACTTGGTGGTTTGCTACG-3′)

*Ifng* (F: 5′-TAACTCAAGTGGCATAGATGTGGAAG-3′,

     R: 5′-GACGCTTATGTTGTTGCTGATGG-3′)

*Gapdh* (F: 5′-CATCGCCTTCCGTGTTCCTA-3′,

     R: 5′-GCGGCACGTCAGATCCA-3′)

### 4.16. Statistical Analysis

Data are expressed as mean ± SD of independent experiments. Significant differences between groups were determined by the Mann–Whitney U test. All statistical analyses were performed with SPSS 11.5 (IBM SPSS, Chicago, IL, USA).

## Figures and Tables

**Table 1:** Determination of the component monosaccharides identified in SH.

No.	Compound	Contents (mg/g Dry Weight)
1	Mannose	1.91
2	Rhamnose	2.13
3	Glucose	-
4	Galactose	8.10
5	Xylose	4.29
6	Fucose	59.49

**Table 2:** Blood parameters in CPA-induced immunosuppressed mice.

	PBS	CPA	SH 50	SH 150
RBC (×10^6^ cells/μL)	11.15 ± 0.22	9.66 ± 0.23 ^#^	10.50 ± 0.23	11.60 ± 0.17 *
HGB (g/dL)	16.6 ± 0.28	14.38 ± 0.33 ^#^	15.6 ± 0.35	17.48 ± 0.25 *
HCT (%)	52.28 ± 1.04	43.93 ± 1.04 ^#^	48.70 ± 1.09	53.85 ± 0.85 *
MCV (fL)	46.92 ± 0.31	45.48 ± 0.14	46.38 ± 0.3	46.43 ± 0.38
MCH (pg)	14.90 ± 0.05	14.88 ± 0.01	14.85 ± 0.07	15.08 ± 0.07
MCHC (g/dL)	31.77 ± 0.18	32.75 ± 0.09	32.02 ± 0.15	32.48 ± 0.13
Monocytes (%)	2 ± 0.20	15.35 ± 1.4 ^#^	11.92 ± 1.3	4.98 ± 1.2 *
Basophils (%)	0.06 ± 0.04	1.34 ± 0.21 ^#^	1.72 ± 0.45	0.53 ± 0.25*
Eosinophils (%)	13.13 ± 0.71	30.83 ± 3.09 ^#^	3.46 ± 0.54 *	3.38 ± 0.79*

RBC, total red blood cells; HGB, hemoglobin; HCT, hematocrit; MCV, mean cell volume; MCH, mean cell hemoglobin; MCHC, mean cell hemoglobin concentration. # *p* < 0.05 vs. naïve mice, * *p* < 0.05 vs. CPA-injected mice.

## Data Availability

The data presented in this study are available upon request from the corresponding author. The data are not publicly available due to an ongoing patent application.

## Supplementary Materials

The following supporting information can be downloaded at: https://www.mdpi.com/article/10.3390/molecules27238253/s1, Table S1: Content of total sugar and sulfate of SH.; Figure S1: SH enhances immune functions in BALB/c mice.; Figure S2: SH increases T cell and neutrophil count in splenocytes.
