# Supplementary material for: Immune-Enhancing Effect of Sargassum horneri on Cyclophosphamide-Induced Immunosuppression in BALB/c Mice and Primary Cultured Splenocytes
Source: Molecules. 2022 Nov 26;27(23):8253. doi: 10.3390/molecules27238253 (PMC9738764; doi:10.3390/molecules27238253)
Supplement: Supplementary file 1 [file molecules-27-08253-s001.zip › molecules-1990064-supplementary.pdf]

Supplementary Materials

Table S1. Content of total sugar and sulfate of SH.

Sample	Total Sugar (%)	Sulfate (%)
Sargassum horneri	39.69	16.93

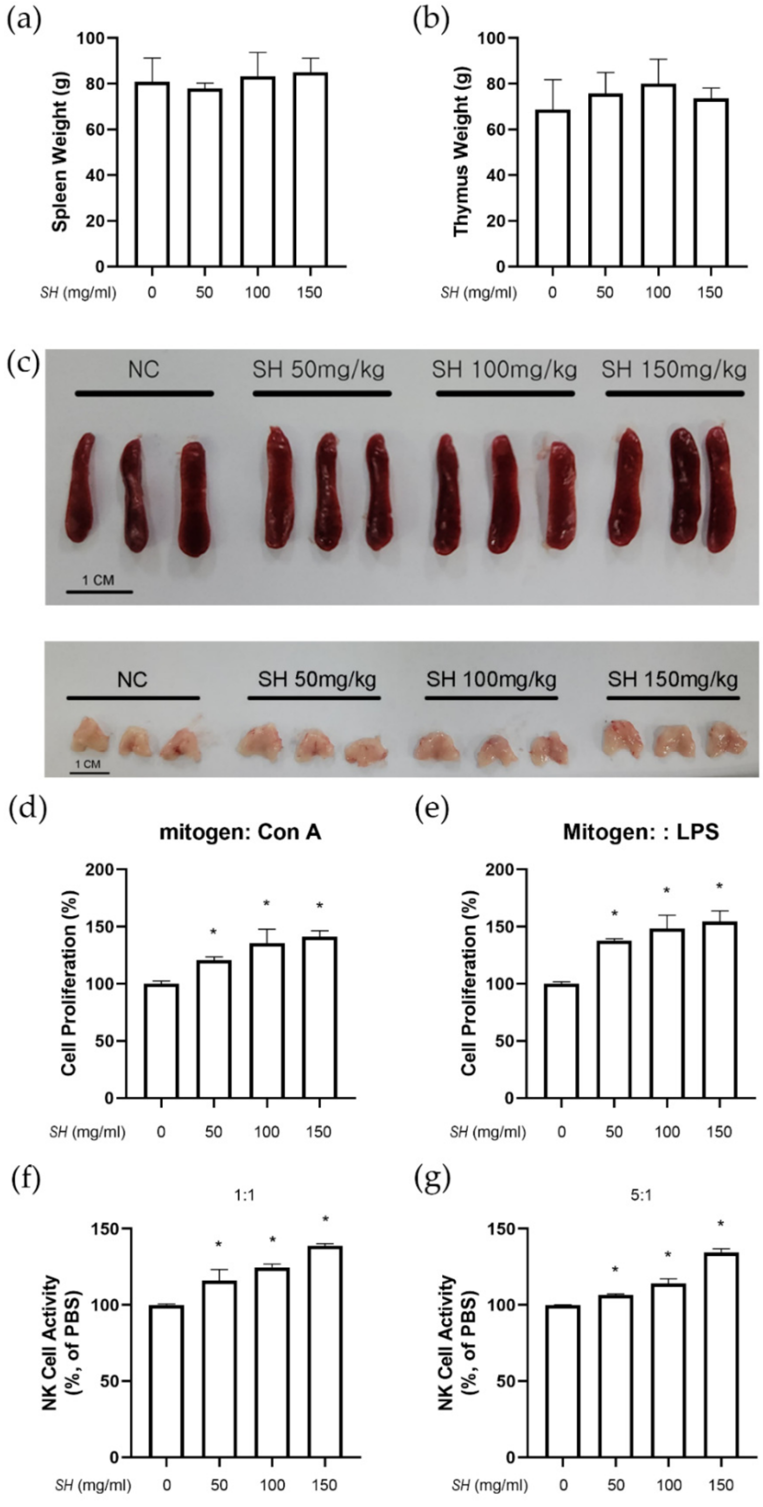

Figure S1. SH enhances immune functions in BALB/c mice. SH was orally administrated in BALB/c mice for 14 days and immune-related parameters were measured. Tissue weight of (**a**) spleen and (**b**) thymus was measured. (**c**) Representative photograph images of spleen and thymus of each group are shown. Splenocytes were isolated from the mice of each group and cell proliferation induction by (**d**) Con A or (**e**) LPS was evaluated. NK cells purified from splenocytes were tested for YAC-1 targeting activities at (**f**) 1:1 and (**g**) 5:1 ratio. *$p < 0.05$ vs. PBS-fed control group. Results are dis-played as mean ± SD of three or more separate experiments.

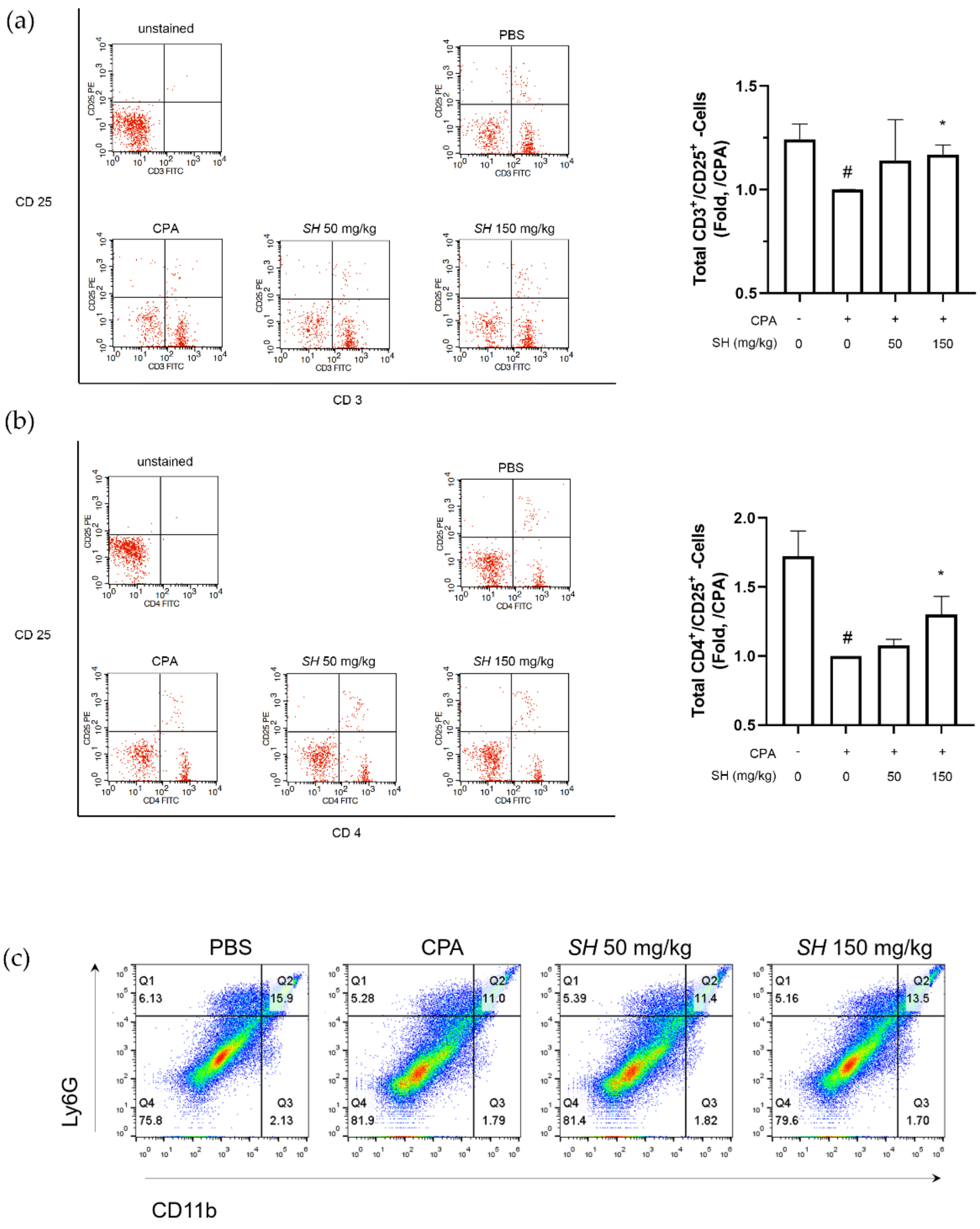

Figure S2. SH increases T cell and neutrophil count in splenocytes. Splenocytes were isolated from the spleen tissue of naïve mice. Flow cytometry was performed to detect (**a**) CD3+/CD25+ T cells, (**b**) CD4+/CD25+ T cells and (**c**) CD11b+/Ly6G+ neutrophils in SH-treated splenocytes.
